# Cost-Effective PEDOT:PSS Temperature Sensors Inkjetted on a Bendable Substrate by a Consumer Printer

**DOI:** 10.3390/polym11050824

**Published:** 2019-05-07

**Authors:** Almudena Rivadeneyra, Marco Bobinger, Andreas Albrecht, Markus Becherer, Paolo Lugli, Aniello Falco, Jose F. Salmerón

**Affiliations:** 1 Pervasive Electronics Advanced Research Laboratory (PEARL), Department of Electronics and Computer Technology, University of Granada, 18071 Granada, Spain; jfsalmeron@ugr.es; 2 Institute for Nanoelectronics, Technical University of Munich, 80333 Munich, Germany; andreas.albrecht@tum.de (A.A.); marco.bobinger@tum.de (M.B.); markus.becherer@tum.de (M.B.); 3 Faculty of Science and Technology, Free University of Bolzano, 39100 Bolzano-Bozen, Italy; paolo.lugli@unibz.it (P.L.); aniello.falco@unibz.it (A.F.)

**Keywords:** inkjet printing, PEDOT:PSS, printed electronics, resistive sensor, silver nanoparticles

## Abstract

In this work, we report on a fabrication protocol to produce fully inkjet-printed temperature sensors on a bendable polyethylene terephthalate (PET) substrate. The sensing layer is made of polymer-based Poly(3,4-ethylenedioxythiophene) polystyrene sulfonate (PEDOT:PSS) ink that is electrically contacted by an underlying interdigitated electrode (IDE) structure based on a silver nanoparticle (AgNP) ink. Both inks are available commercially, and no further ink processing is needed to print them using a cost-effective consumer printer with standard cartridges. The fabricated sensor modules are tested for different IDE dimensions and post-deposition treatments of the AgNP film for their response to a temperature range of 20 to 70 °C and moisture range of 20 to 90% RH (relative humidity). Attributed to the higher initial resistance, sensor modules with a larger electrode spacing of 200 µm show a higher thermal sensitivity that is increased by a factor of 1.8 to 2.2 when compared to sensor modules with a 150 µm-spacing. In all cases, the sensors exhibit high linearity towards temperature and a response comparable to state of the art.

## 1. Introduction

Poly(3,4-ethylenedioxythiophene) polystyrene sulfonate (PEDOT:PSS) is a solution-based organic material that is commercially readily available for many types of applications. It is well-known for its ease-of-processing that allows for depositing it under ambient conditions using scalable deposition methods such as spray coating [1,2], screen printing [3,4], and inkjet printing [5,6]. Further, PEDOT [7,8,9,10], as well as PEDOT:PSS, were shown to be biocompatible [9,11]. So far, PEDOT:PSS has been reported mainly for applications such as i) organic solar cells [5,12], where PEDOT:PSS is used as a hole transport and electron blocking layer, ii) thermoelectric generators [4,13], iii) organic light-emitting diodes (OLEDs) [14,15], touch panels [16,17], paper electronics [18], and organic field-effect transistors (OFETs) [19,20]. Besides these applications, PEDOT:PSS can also be employed as a sensing material to sense humidity [21,22,23,24,25], temperature [24,26], mechanical strain [24], physiological parameters of the human skin [27], drugs [28], cancer biomarkers [29], and gas concentrations [30,31]. However, most of these studies report PEDOT:PSS composites with other functional materials such as carbon nanotubes (CNTs) [26,29], graphene [27,31,32], iron oxide nanoparticles [23], and zinc stannate [22].

In this work, we demonstrate the fabrication of fully inkjet-printed PEDOT:PSS-based humidity and temperature sensors on bendable polyethylene terephthalate (PET) substrates. A thin PEDOT:PSS film is employed as a resistive sensor to detect moisture and temperature in the range of 20–90% RH and 20–70 °C, respectively. Solution-processed silver nanoparticles (AgNPs) were deposited on top of the PEDOT:PSS films as interdigitated electrode (IDE) structures. Besides forming a reliable mechanical and electrical contact to the PEDOT:PSS film, the IDEs also lower the overall resistance of the sensor and increase the reproducibility, which are known advantages [33]. Both films, i.e., the PEDOT:PSS and AgNPs, were deposited using a low-cost and non-dedicated consumer printer (see experimental section). Without any further purification, filtration or dilution, the commercially available inks could be used with the standard cartridges of the consumer printer, reducing substantially the fabrication costs when compared to dedicated inkjet printers. Moreover, no clean room processes are needed, whose facilities costs are drastically high.

The sensitivity of the PEDOT:PSS/AgNP films was studied for different electrode spacings and with respect to different post-deposition treatments of the printed AgNP film before depositing the sensing layer, i.e., no treatment as well as no thermal or photonic sintering. It was found that (i) sensor modules with a larger electrode spacing, and in turn a larger initial resistance and (ii) sensor modules with thermally annealed AgNP films before PEDOT:PSS, exhibit an increased sensor response. The increases in sensitivity were attributed to higher initial resistance and better electrical contact of the PEDOT:PSS and the IDE layer, respectively. In contrast to previous work using composite materials, this contribution shows the sensing capabilities of a fully printed module with a pure PEDOT:PSS layer as the sensing material.

## 2. Materials and Methods

### 2.1. Materials

The silver nanoparticle (AgNP) ink DGP-40LT-15C was purchased from ANP (Korea) and used without purification or processing except for shaking by hand prior to filling the inkjet cartridge. According to the manufacturer datasheet, the AgNP ink has a silver content of 35% and is diluted with the solvent TGME (Triethylene glycol monoethyl ether). The PEDOT:PSS ink was purchased from Sigma Aldrich at a weight content of 1.3 wt. % in deionized (DI) water. In particular, the content of the PEDOT was 0.5 wt. % and that of PSS was 0.8 wt. %. Again, no extra processing was done before filling the cartridge. The substrate employed to define the sensors was PET (product name: Melinex 506) from DuPont® (Wilmington, Delaware, USA).

### 2.2. Fabrication Process

A low-cost consumer inkjet printer, Workforce 2010W from Epson (Japan) with a cost of about 60 € including cartridges, was used without modifications to print the AgNP and PEDOT:PSS films directly to the PET substrate. The black cartridge was replaced by refillable cartridges, which were either filled with the AgNP or the PEDOT:PSS ink.

Before printing the samples, the nozzles were cleaned and it was assured that no nozzles were blocked or misfired because of air within the channels. The samples were printed with the regular printer driver, and the printer was set to the settings Epson Matte, and the quality Strong A fine raster and slow printing were used. The substrate was fed through the built-in paper feed. The drying of the printed layers was done at a temperature of 60 °C for 10 min in a UF55 oven from Memmert (Schwabach, Germany). The photonic sintering of the silver layers was conducted using the Sinteron2010 from Xenon Corporation (USA) at a high voltage of 2.5 kV for a duration of 2 ms. The process parameters for this AgNP ink on the PET substrate were tailored to previous publications [34,35].

We produced three different types of sensors, using the same materials but modifying the fabrication steps to study the influence of the printing process on the sensor performance. The difference among the sensors was the post-deposition treatment of the silver layer when printing the PEDOT:PSS on top, as summarized in Table 1.

### 2.3. Characterization

The thicknesses of the films were measured using a DektakXT® profilometer from Bruker (Billerica, Massachusetts, USA). The sheet resistances were recorded for films with an area of 10 × 10 mm^2^ using a four-point probe setup from Euris GmbH (Munich, Bavaria, Germany) connected to a B2901A Keysight (Santa Rosa, CA, USA) source measuring unit (SMU). For all measurements, a constant current of 100 µA was sourced. 

Scanning electron microscope (SEM) images were recorded with an NVision 40 from Carl Zeiss (Oberkochen, Baden-Wurttemberg, Germany) at an acceleration voltage of 5.0 kV and a working distance of 5.0–5.5 mm.

The electrical measurements were automated with the use of LabVIEW 2016, which controls an impedance analyzer (Keysight E4990A) with an impedance probe kit (42941A) for the sensor readout. The excitation voltage applied in all measurements was V_DC_ = 0 and V_AC_ = 500 mV in the frequency range from 100 Hz to 10 MHz. Calibration was done to compensate for the parasitic elements in agreement with previous work [36,37]. The sensor was placed in a climatic chamber VLC4006) from Vötsch Industrietechnik GmbH (Balingen, Baden-Wuerttemberg, Germany) with temperature and humidity control. The monitoring was performed with the climatic chamber sensor system. For the RH sensing, the moisture content was ramped up in 10% steps and held for 1 h to ensure a stable value in the whole volume of the chamber. For the temperature sensing, a similar approach was used with 5 °C steps for 1 h.

## 3. Results

At first, the sheet resistance and the thickness of the PEDOT:PSS films are characterized and a specific number of the printed PEDOT:PSS layers are selected for the sensor fabrication. The sensor modules are then tested for their response to temperature and moisture and different fabrication flows that, in the following text, are denoted as i) dry, ii) sintered, and iii) wet (see Section 2.2 Fabrication process for more details). The results shown in this paper represent mean values averaged over five different sensor modules that were measured at a frequency of 100 Hz, together with the associated error calculated as the standard deviation of the individual measurement of each sample.

### 3.1. Physical and Electrical Characterization

Before producing the sensors, the printing process of the PEDOT:PSS films was characterized on a PET substrate. In detail, an increasing number of layers was printed and the produced film was measured with respect to its sheet resistance and thickness. The measurements are summarized in Table 2. For the fabrication of the sensor modules, three layers were selected since this number represents the best compromise between reproducible results with a low error and fabrication time.

**Figure 1:**
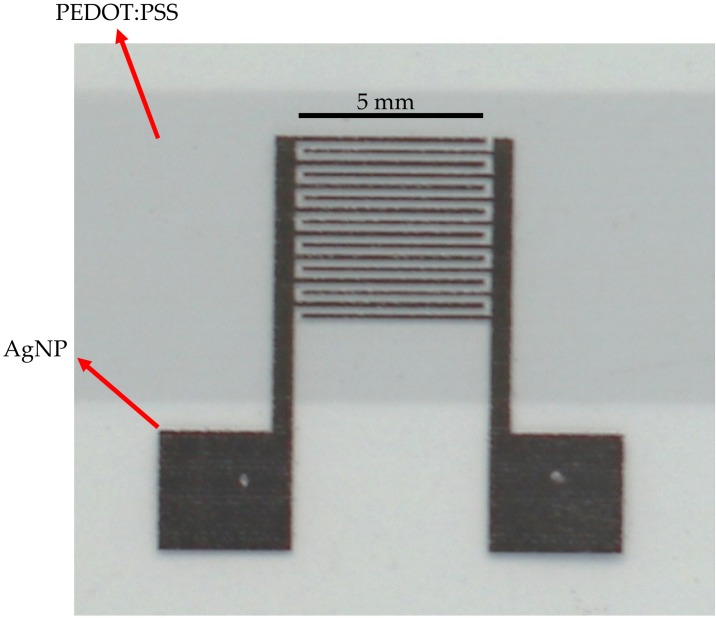
Photograph of a fully inkjet-printed sensor module. The photo was recorded from a dry sensor module (see main text for the description). The AgNP-based electrodes, the PEDOT:PSS sensing layer, and the scale bar are labeled in the image.

**Figure 2:**
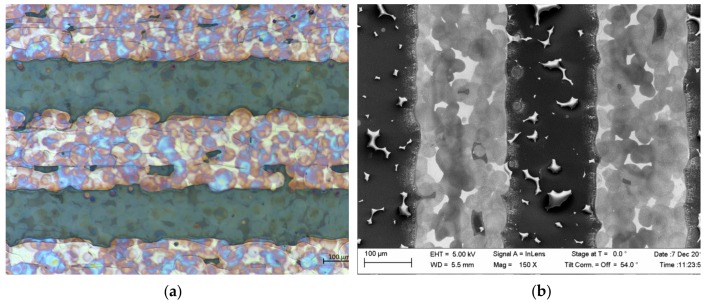
(**a**) Microscope image and (**b**) SEM image for the IDE structure of the printed sensor module shown in Figure 1. For the images, a dried sensor module (see Section 2.2 for more details) was utilized.

A photograph of a dry (see Section 2.2 for the description) sensor module is illustrated in Figure 1. The IDE structure of the AgNP layer is easily distinguishable from the other material, whereas the PEDOT:PSS layer is semitransparent. In the microscope image shown in Figure 2a, it can be seen that the PEDOT:PSS layer covers both the IDE and the PET substrate. The visible blue reflections all over the photograph prove the presence of PEDOT:PSS. The SEM-image depicted in Figure 2a for the dried sample reveals the AgNP-based electrodes as bright areas due to their high conductivity and the PEDOT:PSS-based electrodes as darker areas. The small structures in the sensing channels between the electrodes are charging phenomena that arise due to the non-conductive substrate. We observed no difference in the morphology, the microscope, or the SEM images for the different fabrication flows.

Sensors with varying finger widths and spacings among consecutive fingers were fabricated and characterized to determine the minimum safe resolution that could be used to ensure a high yield rate. Although sensors with low dimensions of 100 µm width and 115 µm spacing can be produced, many of the trials resulted in short circuits. We found a yield rate higher than 85% for the 150 µm width and spacing of 200 µm. For sensors with larger dimensions of 200 µm width and 200 µm spacing, the yield rate was higher than 95%. Therefore, these two dimensions were the ones characterized as temperature sensors.

### 3.2. Temperature Characterization

**Figure 3:**
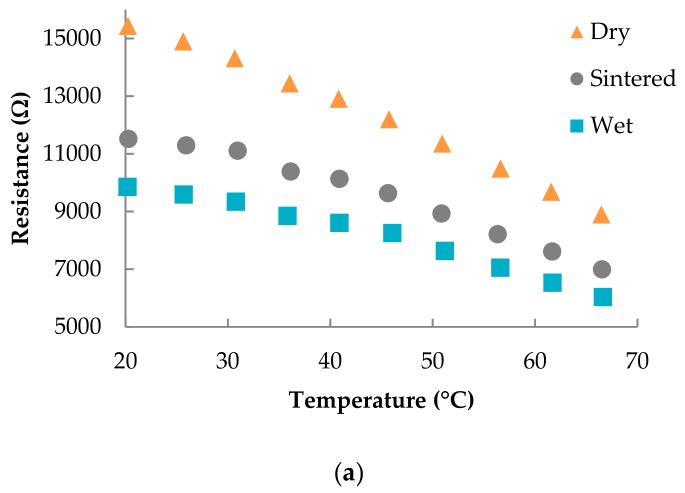

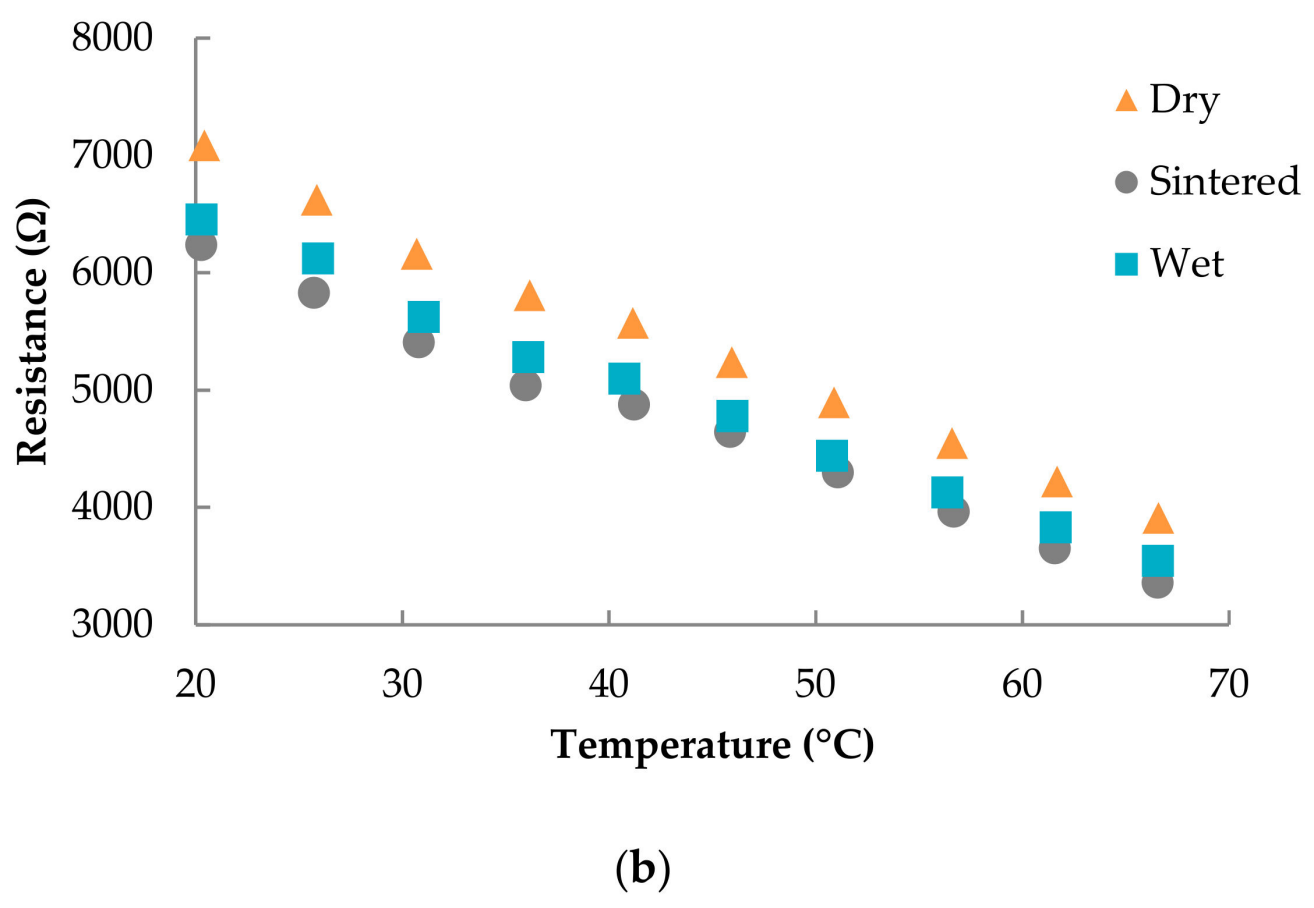
Resistance vs. temperature at 55% RH for electrodes with a width of 150 µm and spacing of (**a**) 200 µm and (**b**) 150 µm. The plots also include the data for different types of process flows; labeled as dry, sintered, and wet (see Section 2.2 for more details).

The different types of sensors exhibited a linear response towards temperature, as can be seen in Figure 3 that illustrates the sensor response for different fabrication flows and electrode spacings of (a) 200 µm and (b) 150 µm. The curves show a clear reduction in sensor resistance with increasing temperature. For both layouts, the dry samples show a higher response than the other two fabrication flows. In the case of wider spacing, sintered samples presented a relatively higher response than the wet ones; whereas both types of sensors had virtually the same behaviour when the spacing among the fingers was reduced.

The fitting curves for the responses to temperature for all the characterized sensors are summarized in Table 3. As commented before, the sensitivity was higher for wider spacing than for the narrow one. For the dry electrodes, we found a sensitivity that was increased by a factor of 2.2 for the 200 µm distance with respect to 150 µm. For the sintered films, this factor was 1.8, and for the wet electrodes, it was 1.4. These results are directly related to the initial resistance of the sensors. For example, the resistance at 20 °C for dry samples is about 15.4 kΩ for a 200 µm distance and about 7.1 kΩ for 150 µm. It can be concluded that the higher the resistance is, the bigger the response of the sensor is. This can be associated with the fact that wider spacing results in a higher volume of sensing element that reacts to the temperature changes.

Further, it was found that sensors made of dried AgNP films before printing the sensing layer (dry) showed higher sensitivities when compared to untreated (wet) or sintered AgNP films. This effect can likely be ascribed to the higher resistance provoked by worse sintering of the AgNP film because of the PEDOT:PSS absorbed part of the energy. The fact that the untreated AgNP film (wet) resulted in lower resistance could be associated with an interaction of the ink solvents. However, it provides a lower but good sensitivity with a simpler and, thus, faster fabrication process than the other two manufactured types of sensors.

**Figure A1:**
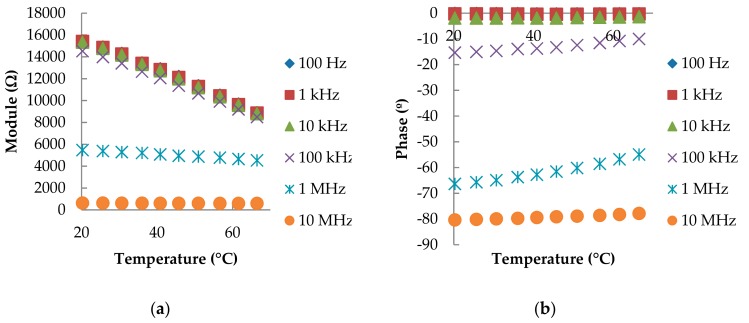
(**a**) Module and (**b**) Phase vs. temperature for the different frequencies of the dry samples.

The variations in the module and phase of the sensors for different working frequencies with respect to temperature (see Appendix A, Figure A1) show that the sensors are purely resistive below a frequency of 100 kHz. Above a frequency of 1 MHz, the sensor is mainly capacitive (phase higher than −70°), indicating that the element seen is only the electrode contribution.

### 3.3. Influence of Moisture Content

**Figure 4:**
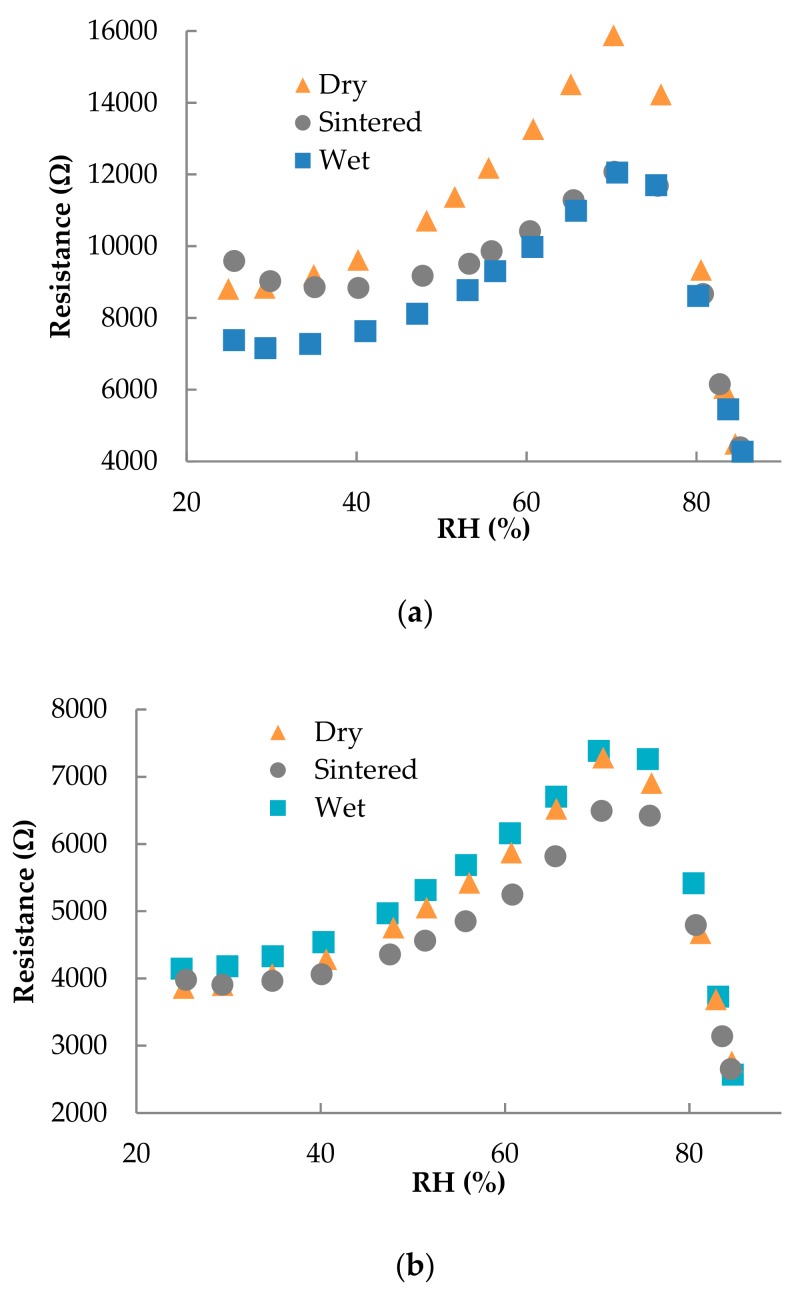
Resistance vs. RH (%) at a temperature of 40 °C for electrodes with a width of 150 µm and spacings of (**a**) 200 µm and (**b**) 150 µm.

We studied the effect of RH on the fabricated sensors since PEDOT:PSS is well-known to be affected by this environmental parameter [38]. Figure 4 depicts the responses of the sensors towards RH for IDE electrodes with a width of 150 µm and spacing of (a) 200 µm and (b) 150 µm. In all cases, the resistance increases from 20% RH to 70% RH, whereas the resistance drastically decreases above 70% RH. The change in the sensor response above 70% RH can be caused by the condensation of water among the electrodes [39,40]. This effect has already been observed by Kus et al. in 2009 [38]. In their work, they studied the change in resistivity of a PEDOT:PSS film up to moisture values of 90% RH. Above a value of 80% RH, similar to our work, they observed a drastic decrease in resistance, which they attributed to the formation of a water meniscus layer on the PEDOT:PSS film, after the water uptake of the film has saturated. After this threshold is reached, the hydrophilic part of the film, composed by the PSS, is attracted to the superficial water meniscus, leaving a PEDOT rich layer on the bottom. Since the former polymer shows an insulating behaviour, its temporary removal can yield a significant reduction of the sheet resistance. Nevertheless, having high humidity content for a prolonged time might compromise the structural integrity of the PEDOT:PSS layer, which could delaminate or partially scratch [41]. Because of these concurring phenomena, the overall effect of RH makes necessary the encapsulation of the sensor to obtain an accurate temperature value, independent on the moisture content in the environment.

**Figure A2:**
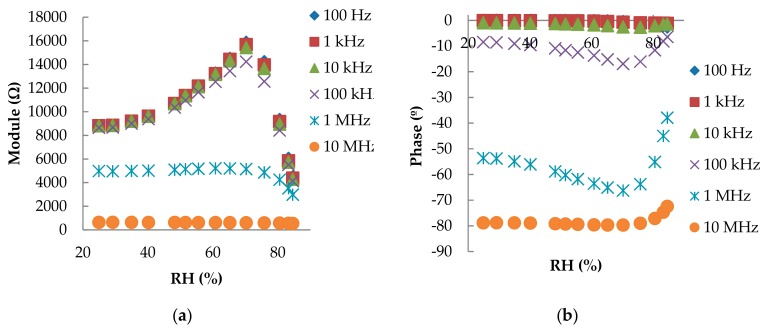
(**a**) Module and (**b**) Phase vs. RH for the different frequencies of the dry samples.

The variations in module and phase of the sensors for different working frequencies with respect to RH are illustrated in Figure A2. Again, the response of the sensors is virtually resistive up to 100 kHz, leading to a capacitive element above 1 MHz.

## 4. Discussion

In this section, the results for the sensor modules are compared and benchmarked within the context of the literature. Table 4 summarizes the performance parameters of various PEDOT:PSS-based sensors along with their deposition technique, substrate material, and composite sensing materials. Previous work has often used composite materials [23,26] or time-consuming and costly fabrication methods that require metal evaporation [38]. Harada et al. have applied a scalable screen printing process to deposit the silver electrodes [26], whereas the PEDOT:PSS film was deposited by hand via a non-scalable and non-reproducible drop-casting process. The hurdle of a lack of scalability and reproducibility of drop-casting processes can be overcome by more advanced techniques that rely on multiple-droplet drop-casting methods [42]. So far, to the best of our knowledge, there exists no work on a fully inkjet-printed sensor that utilizes a pure PEDOT:PSS layer as the sole sensing material. The presented sensors show high humidity and temperature sensitivity of 1.7%/% RH and −0.8%/°C, respectively, that outperforms the values of previous publications [23,24,26]. Nevertheless, one drawback inherent to the PEDOT:PSS-based sensors should not be omitted, i.e., the upper RH limit of around 70 to 80% RH, which is attributed to the formation of a water layer on top of the PEDOT:PSS film when the maximum water uptake is reached. This disadvantage of the PEDOT:PSS sensing films can eventually be overcome by an encapsulation based on graphene oxide or cellulose [43,44].

## Figures and Tables

**Table 1:** Fabrication steps of each of the manufactured sensors.

Type 1: Dry	Type 2: Sintered	Type 3: Wet
Inkjet printing of Ag	Inkjet printing of Ag	Inkjet printing of Ag
Drying of Ag layer	Drying of Ag layer	Inkjet printing of PEDOT:PSS
Inkjet printing of PEDOT:PSS	Sintering of Ag layer	Drying of the sensor ^1^
Drying of the sensor ^1^	Inkjet printing of PEDOT:PSS	Sintering of the sensor ^1^
Sintering of the sensor ^1^	Drying of the sensor ^1^	--

^1^ both Ag and PEDOT:PSS layers.

**Table 2:** Sheet resistance and thickness of 1 cm^2^ sized-squares of plain PEDOT:PSS on a PET substrate, after drying at a temperature of 60 °C for 10 min.

Number of Layers ^1^	Sheet Resistance (Ω/sq.)	Thickness (nm)
1	250 ± 50	41 ± 7
2	170 ± 30	95 ± 9
3	110 ± 10	170 ± 10
4	81 ± 8	260 ± 12
5	29 ± 5	330 ± 15

^1^ Consecutive printing.

**Table 3:** Sensitivity and linearity of the calibration curves shown in Figure 3.

Type of Fabrication	Electrode Separation (µm)	Sensitivity (Ω/°C)	Linearity (R^2^)
Dry	200	−145	0.9968
	150	−66.2	0.9964
Sintered	200	−105	0.9825
	150	−59.7	0.9956
Wet	200	−86.2	0.9845
	150	−61.5	0.9957

**Table 4:** Summary of the relevant process and performance parameters of the PEDOT:PSS-based humidity and temperature sensors.

Ref.	Humidity (Range/Sensitivity)	Temperature (Range/Sensitivity)	Deposition	Substrate	Composition
[23]	30–70% RH 0.625%/%RH	20–50 °C −0.53%/°C	Spin coating	Free-standing	Iron oxide nanoparticles
[26]	X	20–50 °C −0.57%/°C	Screen printing	Polyimide	CNT
[24]	25–95% RH 0.714%/%RH	15–45 °C −0.53%/°C	Dip coating	Fibers	Polyamide fibers
[38]	40–70%RH 6.7%/%RH	X	Drop coating	Glass	pure
This work	20–70% RH 1.7%/%RH	20–70 °C −0.8%/°C	Fully printed	PET	pure
